# GenArk: towards a million UCSC genome browsers

**DOI:** 10.1186/s13059-023-03057-x

**Published:** 2023-10-02

**Authors:** Hiram Clawson, Brian T. Lee, Brian J. Raney, Galt P. Barber, Jonathan Casper, Mark Diekhans, Clay Fischer, Jairo Navarro Gonzalez, Angie S. Hinrichs, Christopher M. Lee, Luis R. Nassar, Gerardo Perez, Brittney Wick, Daniel Schmelter, Matthew L. Speir, Joel Armstrong, Ann S. Zweig, Robert M. Kuhn, Bogdan M. Kirilenko, Michael Hiller, David Haussler, W. James Kent, Maximilian Haeussler

**Affiliations:** 1 Genomics Institute, University of California, Santa Cruz, CA 95064 USA; 2 LOEWE Centre for Translational Biodiversity Genomics, Senckenberganlage 25, 60325 Frankfurt, Germany; 3 Senckenberg Research Institute, Senckenberganlage 25, 60325 Frankfurt, Germany; 4 Institute of Cell Biology and Neuroscience, Faculty of Biosciences, Goethe University Frankfurt, Max-von-Laue-Str. 9, 60438 Frankfurt, Germany

## Abstract

Interactive graphical genome browsers are essential tools in genomics, but they do not contain all the recent genome assemblies. We create Genome Archive (GenArk) collection of UCSC Genome Browsers from NCBI assemblies. Built on our established track hub system, this enables fast visualization of annotations. Assemblies come with gene models, repeat masks, BLAT, and in silico PCR. Users can add annotations via track hubs and custom tracks. We can bulk-import third-party resources, demonstrated with TOGA and Ensembl gene models for hundreds of assemblies.

Three thousand two hundred sixty-nine GenArk assemblies are listed at https://hgdownload.soe.ucsc.edu/hubs/ and can be searched for on the Genome Browser gateway page.

**Supplementary Information:**

The online version contains supplementary material available at 10.1186/s13059-023-03057-x.

## Background

Gone are the days when a new genome for an organism made White House press briefings and news headlines. Instead, several dozen are completed every day and silently submitted to data archives. The number of publicly available genome assemblies from the International Nucleotide Sequence Database Collaboration (INSDC) [1] has reached thousands of animal genomes and millions of bacteria and viruses. In addition, the number is increasing quickly, on average 50% per year for most types of organisms (Additional file 1: Fig. S1), including hundreds of human genomes at unprecedent quality and from diverse populations. At the current growth rate, half a million metazoan (i.e., not bacterial/viral) genomes will be available in 10 years. The ambitious plan of the Earth BioGenome Project [2] to sequence 1.5 million organisms in this timeframe seems within reach.

But to be useful to most life science researchers, the billions of nucleotides will need to be presented via a graphical user interface, not just as raw text files. The existing process of making genome browsers from the raw data and storing them in relational database tables, from selected assemblies based on requests from biologists and then adding and documenting genome annotation tracks to them one-by-one, does not scale. Genome browser teams cannot choose the assemblies that they support on their websites manually anymore, let alone choose appropriate annotation tracks for each species. On the technical side, relational database servers run into practical problems (restarts, backups, table repairs) when the number of databases and tables exceed tens of thousands, so a data store is needed that can handle millions of assemblies and billions of annotation objects. To address these bottlenecks, Ensembl created a Rapid Release platform [3], which contains 1413 genome browsers at the time of writing, and the National Center for Biotechnology Information (NCBI) Genome Data Viewer [4] currently contains around 3000 genomes. But as can be seen from these numbers, most sequenced assemblies are still not yet available in any genome browser.

A different approach to solve the increase in genomes is “crowd-sourcing” the problem to research labs who sequence these genomes. To this end, the UCSC Genome Browser created the assembly track hub system of indexed binary files that, instead of depending on a single centralized relational database, allows any individual lab to create a new genome browser by referencing their own genome and annotations on their own webserver from a text file. Genome sequence and annotations are then streamed on-demand as researchers are browsing this genome. Tools such as G-OnRamp [5] and “MakeHub” [6] make the setup of a such an assembly hub even easier. However, all third-party annotations on these genomes (added by other labs via custom tracks or track hubs [7]) depend completely on the underlying assembly hub and annotations from two different assembly hubs cannot be shown at the same time, as there is no way to assure that the underlying assemblies are identical.

Therefore, while this system allows any third party to build new browsers and others to add annotations to them, data access speed and long-term stability of the underlying assembly hub files are crucial: If the assembly hub is not available anymore or very slow, even temporarily, all track hubs or custom tracks referring to it are affected. Also, beyond the display of annotations, our popular sequence search tool BLAT [8] is essential when working with genomes but requires one powerful server per genome as the entire sequence index needs to be permanently kept in memory. These servers would collectively result in a huge cost in the long term for the groups that run these assembly hubs.

To give the community a stable and fast baseline collection of browsable and searchable assemblies, we modified BLAT and built “GenArk,” a set of assembly hub genome browsers from the NCBI Assembly database [9], currently containing several thousand genomes. They are hosted on our servers and come with basic annotations. Scientists can rapidly browse these genomes, reliably add their own data as custom tracks or track hubs, quickly align sequences with BLAT or primers with in silico PCR, and easily request the addition of other genome assemblies to this collection.

### Results and discussion

In its initial, current version, the GenArk collection already includes 3269 assemblies, https://hgdownload.soe.ucsc.edu/hubs/ stored in roughly 10 terabytes of data. Around 1600 of these can be found in the NCBI GDV and around 600 on the Ensembl Rapid Release website. The GenArk genome browsers cover multiple clades: 159 primates, 409 mammals, 270 birds, 271 fishes, 115 other vertebrates, 598 invertebrates, 554 fungi, and 230 plants. It also includes 446 assemblies from the Vertebrate Genome Project (VGP) and 336 legacy assemblies that have been superseded by newer versions of that organism’s assembly. All 96 currently released human pan-genome assemblies are included. A relational database server is never used to serve these hubs, so the maximum number of assemblies is technically not limited, as file systems can contain many billions of files today. As with UCSC Genome Browsers in the past, we strive to not remove genomes and will retain old assemblies that have been updated by newer ones so that all track hubs and custom tracks continue to work on these.

**Fig. 1:**
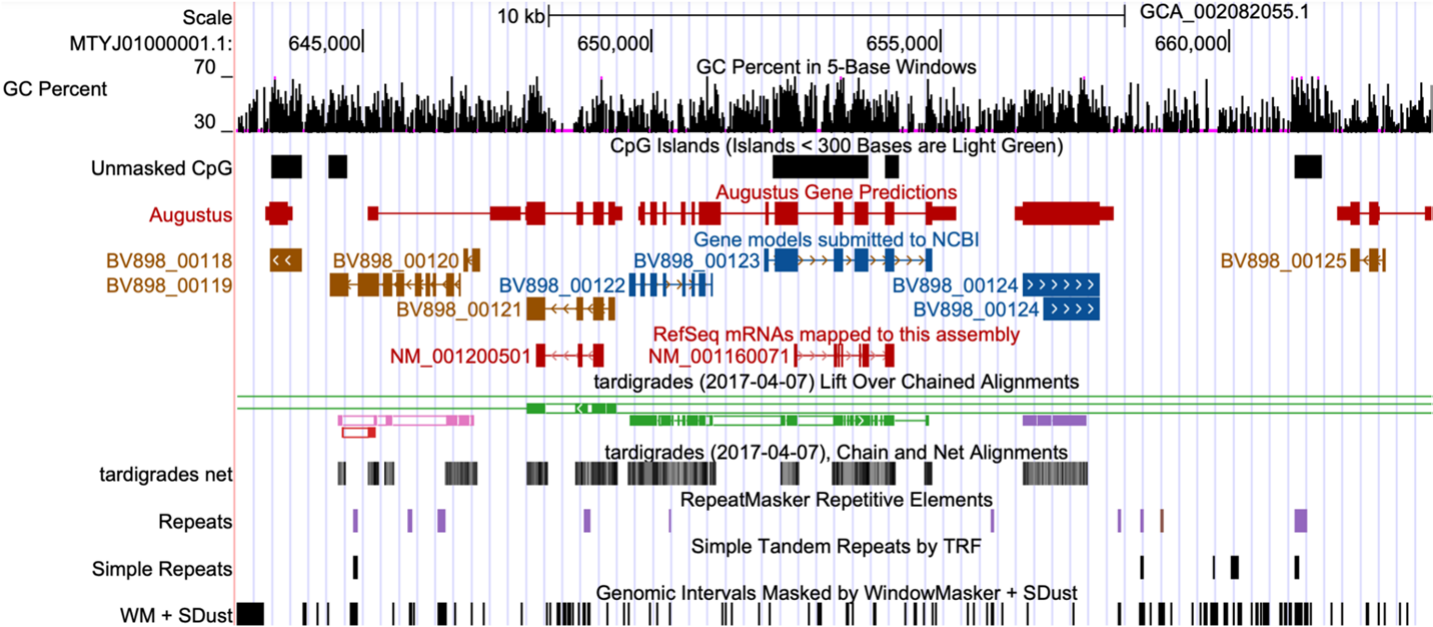
The tardigrade assembly with all standard tracks created by the GenArk annotators. Access this view via its stable session link https://genome.ucsc.edu/s/Max/tardi

All browsers come with a basic set of annotation tracks: “GC Percent,” “CpG Islands,” a “Simple Repeats” track generated with Tandem Repeats Finder, and a RepeatMasker track created from the NCBI annotation files when present or otherwise computed with the RepeatMasker [10] software (Fig. 1).

Beyond repeats, gene models are the most important annotation for researchers. All the transcript tracks discussed in the following use the UCSC bigGenePred format [11] to show codons and amino acids on the genome sequence. For all genomes and as a starting point, basic gene annotations are generated with the AUGUSTUS de novo predictor, using the genome sequence alone. Because the algorithm is run in purely de novo mode, without splice site hints, protein matches or conservation as input, these predictions are not expected to be very accurate and cannot show a name for the gene but give a rough idea of a possible intron/exon transcript structure. To allow human-readable locus name searches for genes, all RefSeq mRNAs from all organisms are aligned to the target assembly using BLAT with a minimum query coverage of 25% and a minimum identity of 35%, to create the “Xeno RefGene” track. For assemblies that have been annotated already by NCBI RefSeq (assembly accessions starting with “GCF”), we build a “NCBI RefSeq” gene model transcript track from the GFF file created by the NCBI Gnomon software [12] which uses a combination of protein matches, RefSeq alignments, and RNA-seq reads from the same species. For GenBank assemblies (accessions starting with “GCA”), if a gene model GFF file was uploaded to NCBI during the submission and is available, we create a transcript track from this file. For either GenBank or RefSeq assemblies, if the genome has also been annotated by Ensembl Rapid Release, we import these transcripts as an “Ensembl” genes track. At the moment, 600 GenArk assemblies have such an Ensembl gene transcript annotation track.

To allow fast sequence searches, we added the new feature “dynamic BLAT” to the BLAT suite. When this feature is activated, the sequence index is stored on disk and loaded on demand into memory as needed. This reduces the required amount of RAM by several orders of magnitude, and as such the cost, as hard disks are around 100 times cheaper than main memory. While a user may experience a potential delay of 20 to 80 s on the first request, subsequent requests are nearly instantaneous. Thanks to this new software feature, we are able to offer BLAT and in silico PCR for all GenArk browsers. At the time of writing, BLAT is still limited to genomes with a maximum chromosome size of 2 Gbp and total genome size of 4 Gbp, so the biggest axolotl chromosome, for example, has to be split into pieces of less than 2 Gbp.

As with all UCSC assemblies, users can add their own annotations using both custom tracks and the more powerful UCSC track hubs. GenArk provides a new sequence name translation system that allows using either NCBI GenBank accessions, NCBI RefSeq accession, or the more familiar sequence names in their UCSC or Ensembl format (chrM=MT, chr1=1, chrY=Y, respectively) for position searches, custom tracks, and track hubs. This means that rewriting of the “sequence name” field of all annotation files is no longer necessary. It also allows annotation files that use the GenBank identifier internally, which means that given a single line of an annotation file, it is always clear what the underlying genome assembly is. Having to guess the correct assembly has been a common problem for decades when sharing annotation files.

For those mammalian genomes where a whole-genome alignment to human or chicken is available, we import TOGA (Tool to infer Orthologs from Genome Alignments) gene models. This method infers orthologous gene loci based on a whole-genome alignment between human and another “query” species, predicts coding exon positions using a hidden Markov model, and classifies transcripts based on whether their reading frame is intact, exhibits inactivating mutations, or lacks exonic sequence due to assembly incompleteness. The special TOGA annotation track provides rich information upon clicking on a predicted transcript. Unlike NCBI RefSeq and Ensembl predictions, this does not require RNA-seq data. Even if genomes have already been annotated with RefSeq/Ensembl gene models, TOGA can detect genes that were missed or mis-annotated by others and additionally provides ortholog inferences. TOGA was run on almost all placental mammals and all bird genomes that were available 1.5 years ago, and we intend to run on all newly sequenced mammals and birds in the future.

Genomes that have not yet been imported can be requested via a new interactive assembly request page at http://genome.ucsc.edu/assemblyRequest.html. If a requested browser is available, a “view” button opens it; otherwise, a “request” button initiates a new browser build; users are notified via email when the process is complete. As with all other genomes, users can save the current browser view with all settings, including the current position, activated tracks, custom tracks, and connected hubs as a short “session” link [13] that can be added to manuscripts via “My Data > My Sessions.”

Many biologists want to see which parts of a genome are conserved, which requires a pairwise or multiple alignment of entire genomes to each other. For computational researchers, an alignment allows mapping annotation locations between genomes using our tool “liftOver” [14]. These pairwise alignments can be requested from our support email address manually, indicating the NCBI accessions of the pair of assemblies desired. We have received 39 requests of this type to date and have added the resulting alignments and liftOver chain files. As time and scheduling permits, these requests can usually be calculated within 1–3 days for the respective GenArk browsers.

#### Example of making a GenArk request

The following exemplifies how to access and request a GenArk assembly hub. A user visits the Genome Browser Gateway page, https://genome.ucsc.edu/cgi-bin/hgGateway, and enters “rabbit” in the “Enter species” box. If the genome is available, a drop-down menu shows “rabbit (Thorbecke 2009 Broad RefSeq) GCF_000003625.3” (Additional file 1: Fig. S2). Clicking this entry launches its Genome Browser.

If this is not the rabbit genome desired, however, the user can click “Unable to find a genome?” link to an FAQ which links to the “assembly request page”: http://genome.ucsc.edu/assemblyRequest.html (Additional file 1: Fig. S3). On the page, one can search for “rabbit.” Only the first 500 assemblies of nearly 15,000 are shown; more can be shown with “show all” under “select assembly type to display.” Next to the New Zealand white rabbit GCA_009806435.2_UM_NZW_1.0, there is a “request” button. The user can enter their email address and indicate the accession number of another assembly in the comment box if a whole-genome alignment is needed (Additional file 1: Fig. S4).

Scientists sequencing new organisms can deposit their genome in GenBank and then contact UCSC to expedite adding their organism to the GenArk collection. Read the first GenArk blog post for a real-world example involving a novel zebrafish genome: https://genome-blog.soe.ucsc.edu/blog/2021/11/23/genark-hubs-part-1/.

## Conclusions

Many smaller research communities now have a genome assembly for their organism but lack the resources of the model species to set up a website with an annotation database and BLAST servers. For these, our GenArk browsers provide a starting point that can be easily extended by the community itself via track hubs. Unlike some assembly hubs built by individual researchers, our GenArk hubs provide a stable set of genome browsers in a consistent format on a fast server that will not move to other institutions nor disappear in the foreseeable future. Freed from the constraints of a single central database server, our system will be able to handle an extremely high number of genome browsers in the future, including the incoming wave of high-quality human genome assemblies.

Given that our average animal RefSeq genome browser size is 1.3 GB (without the BLAT indexes and a copy of the FASTA file), 1 million browsers would take up only 1.3 PB. Therefore, if past improvements of hard disk prices hold (14$/TB in 2022, a x3.5 improvement over the last 10 years), storage cost will not be prohibitively high even for several million genomes, especially compared to the sequencing cost.

Our new, on-demand BLAT servers are slower for the first request than the permanent BLAT servers that we offer for the major model organisms but faster than disk-based BLAST searches. Additional collections of annotation tracks can be added to GenArk browsers in the future, TOGA and Ensembl genes are only the first instances of such an annotation type, and readers are encouraged to send us suggestions on other annotation resources. We hope to identify and add similar cross-organism annotation resources in the future, for example, to make tracks with protein annotations and orthology information. To our delight, the genome browser IGV [15] added support for GenArk assembly hubs as this article went to press, and the Jbrowse2 [16] authors are planning to add support for assembly hubs very soon (pers. comm. Ian Holmes). We hope that our GenArk hubs will help researchers studying the new diversity of human genomes or organisms beyond the common models, to make optimal use of all these data, independent of the genome browser used, first for the thousands of genomes available today and later for the millions of genomes produced by sequencers over the next decades.

## Methods

GenArk hubs are created from the database NCBI Assembly, which contains tens of thousands of genomes submitted by sequencing centers to INSDC databases worldwide. Given the NCBI Assembly FTP directory structure, scripts convert the data into a set of files that form an “assembly hub,” a collection of binary indexed files, usually one per annotation track, described by plain text files. All conversions are written in Perl/shell/python scripts use the UCSC Parasol cluster job scheduler (https://genecats.gi.ucsc.edu/eng/parasol.html), execute Genome Browser command-line tools (https://github.com/ucscGenomeBrowser/kent). The primary driver script in this repository is src/hg/utils/automation/doAssemblyHub.pl. It contains one step per track: Assembly Gaps, a Cytoband diagram, GC Content, RepeatMasker, Simple Repeats [17], WindowMasker [18], the FASTA Softmask, Gaps, Tandem duplicates, CpG Islands, “submitted gene models” for GenBank assemblies, RefSeq genes for RefSeq [19] assemblies, Xeno RefGene (see below), and Augustus-predicted genes [20]. Additional steps, unrelated to annotation tracks, create the genome description HTML page, the softmasked FASTA genome file, checksums for all sequences, and the “trackDb” configuration file that defines the display parameters for tracks.

The scripts also generate a pre-computed BLAT index, used to launch dynamic BLAT and PCR services, which is a memory-mapped index stored on disk (see description below). For selected genomes, we can add third-party annotations to all applicable genome browsers directly, which we demonstrate here with TOGA [21] gene models for around 1000 bird and mammal assemblies.

### Supplementary Information

**Additional file 1:** **Supplemental Figures 1-4.**

**Additional file 2.** Review history.

## Data Availability

The data for all 2430 GenArk assemblies can be downloaded from [22]. The source code required to build the GenArk hubs is available from GitHub under the MIT license [23]. The version from September 2023 has been deposited in Zenodo under DOI:10.5281/zenodo.8321684 [24]. Software tools and source code are also available from https://hgdownload.soe.ucsc.edu/ and via various package managers and software distribution systems (conda, docker, and binary downloads for OSX and Linux).
