# Supplementary material for: GenArk: towards a million UCSC genome browsers
Source: Genome Biol. 2023 Oct 2;24:217. doi: 10.1186/s13059-023-03057-x (PMC10544498; doi:10.1186/s13059-023-03057-x)
Supplement: Supplementary file 1 — Additional file 1: Supplemental Figures 1-4. [file 13059_2023_3057_MOESM1_ESM.docx]

**Supplementary figures:**

- - - 1. Growth of completed assemblies
      2. Assembly search on gateway page
      3. Assembly request page
      4. Assembly request form


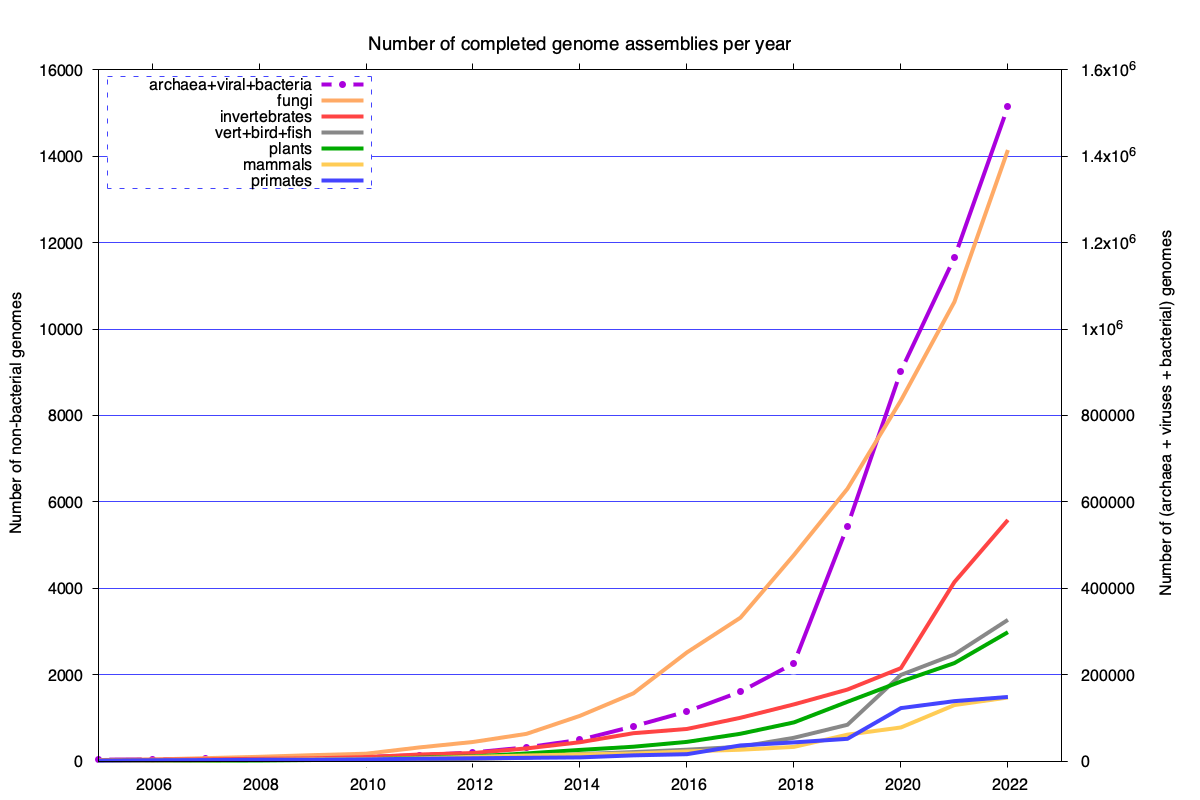


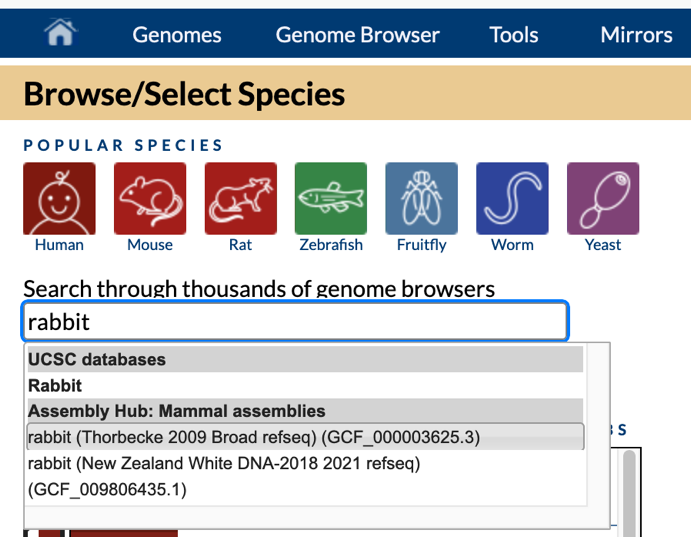


Supplemental Figure 2: Genome search form on UCSC genome gateway page showing the results for the query “rabbit”.


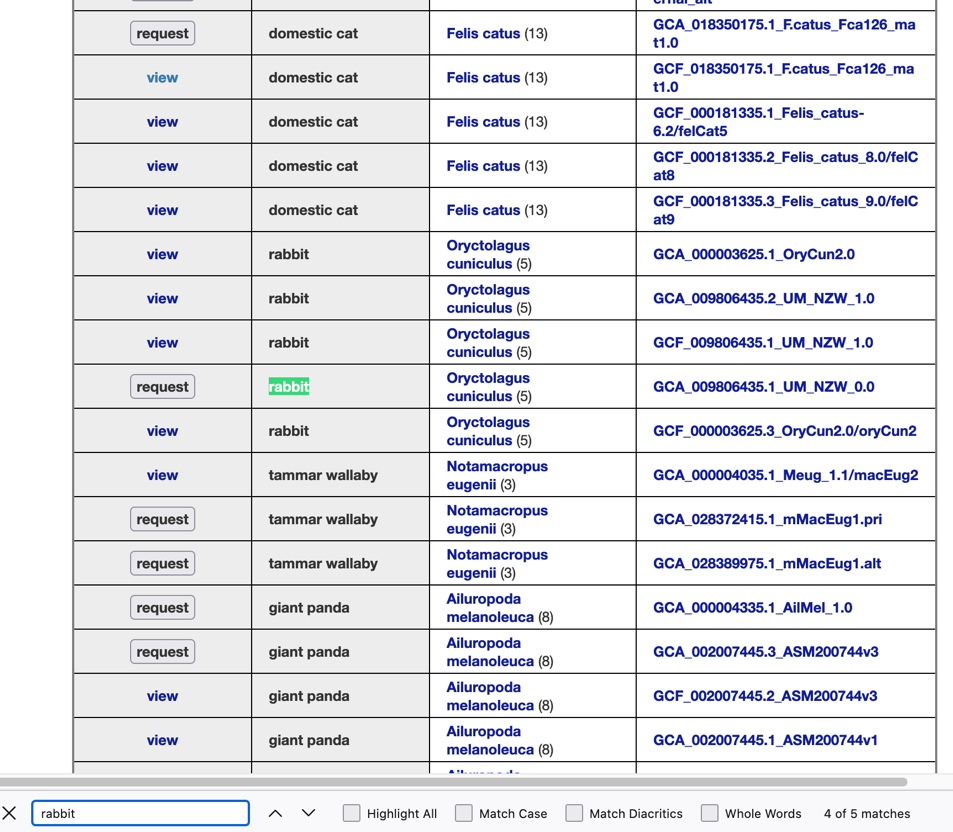


Supplemental Figure 3: Screenshot of the assembly request page, with a search for “rabbit”


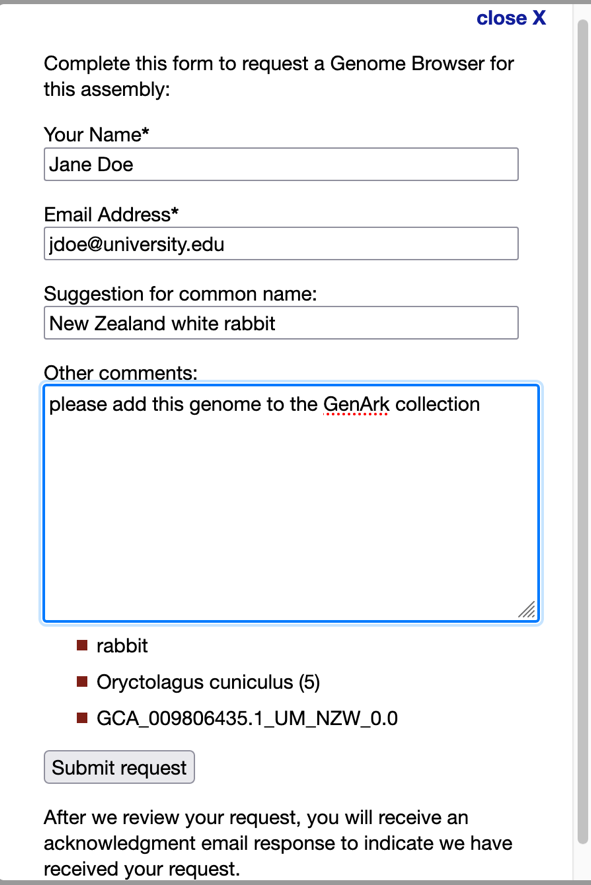


Supplementary Figure 4: Assembly request form
