# Supplementary material for: GenArk: towards a million UCSC genome browsers
Source: Genome Biol. 2023 Oct 2;24:217. doi: 10.1186/s13059-023-03057-x (PMC10544498; doi:10.1186/s13059-023-03057-x)
Supplement: Supplementary file 2 — Additional file 2. Review history. [file 13059_2023_3057_MOESM2_ESM.docx]

Review History

**First round of review**

**Reviewer 1**

**Were you able to assess all statistics in the manuscript, including the appropriateness of statistical tests used?**

There are no statistics in the manuscript.

**Comments to author:**

The manuscript "GenArk: Towards a million UCSC Genome Browsers" describes a long standing resource from the UCSC Genome Browser group and provides access to over 2,400 genome assemblies from NCBI. Said genomes are available from the UCSC Genome Browser web page where it is very straightforward to navigate through a high level taxonomic grouping and quickly into a tabular summary of common name, RefSeq assembly accession and BioSample and BioProject identifiers. Genomes are presented with either their GCF and GCA. I assume this is dependent upon the genome is annotated by RefSeq or not. The menu also describes significant improvements in the BLAT alignment tool which enables index reuse and resolves a long-standing challenge of BLAT needing a persistent server for fast alignments.

Overall I feel the manuscript is excellent and describes powerful tool for researchers to use to further their investigations and provide additional functionality to genomes, a number of which are unannotated by either RefSeq or Ensembl. There are three comments I have about the resource in general and the manuscript but these should not be considered a reflection of the work done as I believe it is excellent. These are detailed below.

1. Understanding the numbers of UCSC/Augustus annotated genomes vs RefSeq annotated genomes

The manuscript mentions the number of genomes available via GenArk as 2,430 and then cites NCBI Genome Data Viewer (GDV) containing 1,690 genomes and Ensembl Rapid Release containing 1,202 genomes. What is not clear is what proportion of the 2,430 hosted genomes are available in these other resources. Is it reasonable to assume that NCBI will host a genome in GDV and therefore UCSC has provided first pass annotation of 740 genomes? Can the authors clarify this please.

2. Import of annotation other than RefSeq

The manuscript clearly describes the level of support available for the imported genomes. However scanning through the hosted lists it becomes clear that there is overlap between genomes which are unannotated by RefSeq but are annotated by Ensembl. Considering Ensembl annotation will either be based on transcriptomic data or results from the BRAKER2 tool there is a real opportunity to improve GenArk's offerings. Even a possibility to provide a merged annotation set from both projects.

For example, the assembly for the moth Abrostola tripartita (ilAbrTrip1.1, GCA_905340225.1) is made available through the GenArk infrastructure. However it has only received Augustus and RefSeq mRNA alignments. The Ensembl project has annotated this genome with downloads available from https://ftp.ensembl.org/pub/rapid-release/species/Abrostola_tripartita/GCA_905340225.1/ensembl/geneset/ in standard file formats.

Do the authors have plans to import Ensembl annotation?

3. Application of TOGA on hosted genomes and wider applicability

The manuscript mentions TOGA gene models were generated "for around 1000 bird and mammal assemblies". What is the criteria used to use TOGA on a GenArk hosted assembly? Are there limits around taxonomic applicability? Do you only run TOGA on genomes with RefSeq annotation? Can the authors clarify this please.

**Reviewer 2**

**Were you able to assess all statistics in the manuscript, including the appropriateness of statistical tests used?**

There are no statistics in the manuscript.

**Comments to author:**

This is a fairly straight forward release of a new tool. The tool appears to be fairly simple to use and the links to various components are indicated. I would only suggest perhaps the addition of screen shots running through the rabbit example to improve clarity and intuitiveness for the tool.

**Authors Response**

**Point-by-point responses to the reviewers’ comments:**

Reviewer #1:

Genomes are presented with either their GCF and GCA. I assume this is dependent upon the genome is annotated by RefSeq or not

*Reply: Yes. The manuscript mentions these prefixes: “For assemblies that have been annotated already by NCBI RefSeq (assembly accessions starting with “GCF”), we … For GenBank assemblies (accessions starting with “GCA”), we … ”*

The manuscript mentions the number of genomes available via GenArk as 2,430 and then cites NCBI Genome Data Viewer (GDV) containing 1,690 genomes and Ensembl Rapid Release containing 1,202 genomes. What is not clear is what proportion of the 2,430 hosted genomes are available in these other resources. Is it reasonable to assume that NCBI will host a genome in GDV and therefore UCSC has provided first pass annotation of 740 genomes? Can the authors clarify this please.

*Reply: Good question. We updated the numbers: As of early June 2023, there are approximately 3,200 assemblies in GenArk, 3,000 assemblies in the NCBI GDV system. Doing the intersection, we see that there are approximately 1,600 assemblies in common between the two systems. We are unaware of the exact selection criteria for including an assembly in the NCBI GCF system, NCBI told us in a phone call that they require at least one RNA-seq experiment in the SRA and that they prefer assemblies from certain projects and prefer mammalian assemblies over invertebrates. We can state that the selection criteria for an assembly to be included in the GenArk collection is to stay current with updated RefSeq/GCF assemblies from NCBI, and add any GenBank/GCA assemblies as requested by users or that are being used in sequencing projects that we are coordinating our resources with those projects. Put differently, we will keep adding RefSeq-annotated assemblies to GenArk, will always add Genbank assemblies that were requested by users and will also add assemblies referenced in the human assembly, e.g. assemblies used for the 470 vertebrate alignment in hg38.*

Manuscript changes: Updated all assembly counts in manuscript. Added to manuscript in results section: “Around 1600 of these can be found in the NCBI GDV and around 600 on the Ensembl Rapid Release website.”. Also added Ensembl as another source of cross-genome annotations, beyond TOGA in the discussion section.

*Reply: The manuscript clearly describes the level of support available for the imported genomes. However scanning through the hosted lists it becomes clear that there is overlap between genomes which are unannotated by RefSeq but are annotated by Ensembl. Considering Ensembl annotation will either be based on transcriptomic data or results from the BRAKER2 tool there is a real opportunity to improve GenArk's offerings. Even a possibility to provide a merged annotation set from both projects.*

For example, the assembly for the moth Abrostola tripartita (ilAbrTrip1.1, GCA_905340225.1) is made available through the GenArk infrastructure. However it has only received Augustus and RefSeq mRNA alignments. The Ensembl project has annotated this genome with downloads available from

<https://ftp.ensembl.org/pub/rapid-release/species/Abrostola_tripartita/GCA_905340225.1/ensembl/geneset/> in standard file formats.

Do the authors have plans to import Ensembl annotation?

*Reply: Excellent suggestion. As indicated above, we identified over 600 genome assemblies in common between the GenArk genomes (>3,200 genomes) and the set of Ensembl Rapid Release genomes (>1,400 genomes). We have now imported the Ensembl gene annotations for these 600 assemblies to our GenArk genome browsers now. We will keep adding Ensembl gene transcript models when they are available in the future as part of the standard GenArk build process.*

*Manuscript changes: Added to results section: “For either GenBank or RefSeq assemblies, if the genome has also been annotated by Ensembl Rapid Release, we import these transcripts as an “Ensembl” genes track. Currently 600 assemblies have such an Ensembl gene transcript annotation track”*

3. Application of TOGA on hosted genomes and wider applicability The manuscript mentions TOGA gene models were generated "for around 1000 bird and mammal assemblies". What is the criteria used to use TOGA on a GenArk hosted assembly?

*Reply: TOGA uses a whole genome alignment to a ‘reference’ genome that provides a gene annotation and infers and annotates orthologous genes. Since TOGA can detect fragments of orthologous genes, it can be run on any assembly (even highly fragmented ones that comprise tens of thousands of smaller scaffolds). We demonstrated this in the TOGA publication (now published https://www.science.org/doi/abs/10.1126/science.abn3107) for pretty much all placental mammals and all bird genomes that were available ~1.5 years ago. Since most of these assemblies lack a comprehensive gene annotation (in particular those that lack transcriptomics data will not be annotated by Ensembl or NCBI), we provided the TOGA annotations of these birds and mammals in GenArk to facilitate analyzing these genomes.*

*Since we automated TOGA’s application and since human/mouse and chicken provide a high-quality annotation (a basis for TOGA), we intend to run TOGA on new mammals and birds that will be sequenced in future and make these annotations available via GenArk.*

*Manuscript changes: Updated preprint reference in manuscript to Science journal. Added sentence in TOGA section: “Unlike NCBI RefSeq and Ensembl predictions, this does not require RNA-seq data. Even if genomes have been annotated with gene models, TOGA can detect genes that were missed or mis-annotated by others, and additionally provides ortholog inferences. TOGA was run on almost all placental mammals and all bird genomes that were available 1.5 years ago and we intend to run on all newly sequenced mammals and birds in the future.“*

Are there limits around taxonomic applicability?

*Reply: TOGA uses alignments between intronic and intergenic regions to infer orthologous gene loci. Hence, evolutionary distance between the species with the new assembly and the reference species is a key criterion, but this does not limit TOGA’s applicability. Based on our experience, we recommend evolutionary distances of up to ~0.6 substitutions per neutral site. This largely holds when using human or mouse as a reference to annotate other placental mammals, or when using chicken as a reference for other birds. TOGA was also tested on other vertebrate groups (e.g. it works well for turtles, reptiles), lepidoptera, sea urchins, and ants. While there is certainly room for optimizing TOGA to non-vertebrate clades, we believe the only factor that restricts TOGA’s taxonomic applicability is the availability of a well annotated reference species at an appropriate evolutionary distance.*

*Manuscript changes: See above.*

Do you only run TOGA on genomes with RefSeq annotation? Can the authors clarify this please.

*Reply: We run TOGA on genomes with or without a RefSeq annotation. For those that lack a RefSeq annotation, TOGA provides a high-quality annotation of coding genes. As we show in the TOGA paper, there is also value in running TOGA for RefSeq-annotated genomes, as TOGA can detect genes that were missed or mis-annotated by RefSeq, and TOGA additionally provides ortholog inferences.*

*Manuscript changes: See above.*

Reviewer #2:

This is a fairly straight forward release of a new tool. The tool appears to be fairly simple to use and the links to various components are indicated. I would only suggest perhaps the addition of screen shots running through the rabbit example to improve clarity and intuitiveness for the tool.

*Reply: Excellent idea, thanks.*

*Changes to manuscript: Added additional supplemental figure 2-4 with the three screenshots of the rabbit example.*

**Second round of review**

**Reviewer 1**

The authors have answered all questions in full and provided appropriate clarifications to the manuscript.

**Reviewer 2**

All suggested changes have been made.
